# Do parental cognitions during pregnancy predict bonding after birth in a low-risk sample?

**DOI:** 10.3389/fpsyg.2022.986757

**Published:** 2022-11-14

**Authors:** Agnes Bohne, Dag Nordahl, Ragnhild Sørensen Høifødt, Vibeke Moe, Inger Pauline Landsem, Catharina E. A. Wang, Gerit Pfuhl

**Affiliations:** ^1^Department of Psychology, Faculty of Health Sciences, UiT The Arctic University of Norway, Tromsø, Norway; ^2^Division of Child and Adolescent Health, University Hospital of Northern Norway, Tromsø, Norway; ^3^Department of psychology, University of Oslo, Oslo, Norway; ^4^Department of Health Care Sciences, Faculty of Health Sciences, UiT The Arctic University of Norway, Tromsø, Norway; ^5^Department of Psychology, Norwegian University of Science and Technology, Trondheim, Norway

**Keywords:** bonding, repetitive negative thinking, infant temperament, perinatal depression, implicit associations, attentional bias

## Abstract

Parental bonding to their infant is important for healthy parent-infant interaction and infant development. Characteristics in the parents affect how they bond to their newborn. Parental cognitions such as repetitive negative thinking, a thinking style associated with mental health issues, and cognitive dispositions, e.g., mood-congruent attentional bias or negative implicit attitudes to infants, might affect bonding.

To assess the influence of cognitive factors on bonding, 350 participants (220 pregnant women and their partners) were recruited over two years by midwives at the hospital and in the communal health care services. Participants were followed throughout the pregnancy and until the infant was seven months old as a part of the Northern Babies Longitudinal Study. Both mothers and fathers took part. First, we measured demographics, repetitive negative thinking, attentional bias, and implicit attitudes to infants during pregnancy, as predictors of bonding two months postnatally. Second, we also measured infant regulatory problems, and depressive symptoms at two months postnatally as predictors of parents’ perception of infant temperament at five months. Robust regression analyses were performed to test hypotheses.

Results showed that mothers and fathers differed on several variables. Parity was beneficial for bonding in mothers but not for fathers. Higher levels of mothers’ repetitive negative thinking during pregnancy predicted weaker bonding, which was a non-significant trend in fathers. For fathers, higher education predicted weaker bonding, but not for mothers. Mothers’ perception of their infant temperament at five months was significantly affected by bonding at two months, but for fathers, their depressive symptoms were the only significant predictor of perceived infant temperament.

In conclusion, for mothers, their relationship with their infant is essential for how they experience their infant, while for fathers their own wellbeing might be the most important factor. Health care providers should screen parents’ thoughts and emotions already during pregnancy to help facilitate optimal bonding.

## Introduction

Bonding is defined as the emotional tie from a parent to the infant (Bicking Kinsey and Hupcey, 2013). It refers to the affective component of the parent’s relationship to the infant, the emotions, and feelings towards the infant. Low quality of bonding may negatively affect parenting behavior, as it could lead to less positive maternal feelings and more irritability and hostility towards the infant (Brockington, 2004; Bicking Kinsey and Hupcey, 2013). Poor bonding is also related to parents’ well-being, as it predicts parenting stress (de Cock et al., 2017), and parenting stress affects child development (Barroso et al., 2018; Fredriksen et al., 2018). Overall, bonding quality is positively related to the infant’s developmental outcomes (Mason et al., 2011; Alhusen et al., 2013; de Cock et al., 2017; Le Bas et al., 2020). Identifying potential precursors of bonding quality can inform how to mitigate poor bonding and parenting stress.

### Predictors of maternal bonding

Previous research has investigated both demographic factors and parental mental health as possible predictors of bonding, with varying results. Kinsey et al. (2014) found a negative effect of socioeconomic status on the quality of bonding, where mothers who were less educated, had lower income, and were less likely to be married reported higher levels of bonding. Cuijlits et al. (2019) also reported a negative effect of education on prenatal bonding in mothers, but not on postnatal bonding. Prenatal maternal depressive symptoms are negatively associated with bonding after birth (Dubber et al., 2015; Rossen et al., 2016; Cuijlits et al., 2019), while prenatal anxiety is not (Dubber et al., 2015; Rossen et al., 2016). As bonding includes forming a relationship to one’s infant, maternal relational experiences have been examined. Mothers’ own attachment style is related to both prenatal (Alhusen et al., 2013) and postnatal maternal–infant bonding (Nordahl et al., 2020). Similarly, Nordahl et al. (2019) also investigated mothers’ early maladaptive schemas, which are negative and stable self-assumptions about oneself and one’s relationships, and found they were negatively associated with prenatal maternal bonding. This indicates that parents’ predispositions in the form of cognitive and relational styles can affect bonding to their child, maybe even to a higher degree than mental health in general.

#### Bonding and parental cognitions

The adverse effects of maternal mental illness on infant development is well established (Goodman et al., 2011; Kingston et al., 2012; Stein et al., 2014). Maternal cognitions or preoccupations might explain this relation (Stein et al., 2009, 2012). Indeed, the tendency to be caught up in negative thoughts during pregnancy is associated with mother-infant bonding after birth (Müller et al., 2013; Schmidt et al., 2017). Emotional availability in the parent is important in the formation of the parent-infant bond (Bicking Kinsey and Hupcey, 2013), accordingly, preoccupied parents might struggle more with bonding. In a model by DeJong et al. (2016) repetitive negative thinking, combined with reduced cognitive control and cognitive biases, occupies mental capacity and leads to less parental sensitivity. In turn, this can cause parents to miss out on positive infant cues, interpret cues more negatively, and cause the infant to either become more passive from the lack of response or express more negative emotionality to get attention. This might facilitate difficulties in parent-infant bonding, parent-infant interaction, and how the parents perceive their infant’s temperament.

Although there is some evidence of a negative effect of repetitive negative thinking on maternal bonding, less is known about other cognitions. Attentional bias has received a considerable amount of attention in depression research. Depressed individuals tend to engage more with sad stimuli than healthy controls, a mood-congruent attentional bias (Gotlib and Joormann, 2010). This tendency was also found in expecting parents with depressive symptoms when looking at emotional infant faces (Bohne et al., 2021). Parents who are caught up in their infant’s sad expressions might experience their child as having more negative emotionality than other parents. Attentional bias towards sad faces may thereby interfere with an optimal bonding process.

Implicit attitudes are predictive of behavior (Greenwald et al., 2009), particularly when under stress and being low on self-regulatory resources. Negative implicit attitudes to infants could thus affect parenting behavior and possibly parents’ emotions towards their infant. For example, Sun et al. (2021) demonstrated that pregnant women’s reported attitudes towards infant crying were not related to their implicit attitudes to the sounds of infant crying. Less is known whether these implicit attitudes affect bonding.

### Parental perception of infant temperament

The infants’ emotionality and regularity, and if they are experienced as easy or difficult, are often referred to as infant temperament (Rothbart and Putnam, 2002). Parents’ report of infant temperament is their subjective experience and, therefore, might be colored by the parents’ cognitions and well-being (Davies et al., 2021). Accordingly, studies found that caregivers’ perception of infant crying as problematic was not related to the actual amount of crying (van der Wal et al., 1998; MacKenzie and McDonough, 2009). However, the infant itself is an active part in every parent-infant relationship, and actual infant behavior might also affect both bonding and parental perception of their infant (Bicking Kinsey and Hupcey, 2013; de Cock et al., 2016). It may be harder to connect to an infant that cries a lot, or has sleep difficulties, as this might be experienced as exhausting and challenging for the parents. Therefore, when parents report difficulty with their infant’s emotional regulation, this could be an expression of both actual regulatory problems in the infant and parental perception of the infant.

### Differences between paternal and maternal bonding

Traditionally, research on parent-infant bonding has focused on mothers, though recent studies investigated father-infant bonding (de Cock et al., 2016, 2017; Scism and Cobb, 2017; Bieleninik et al., 2021). As maternal bonding, paternal bonding is associated with child development (Ramchandani et al., 2013; de Cock et al., 2017) and bonding patterns are similar between mothers and fathers. However, fathers are more likely to have high levels of bonding to their firstborn child than to later-born children, which is not the case in mothers (de Cock et al., 2016). Like mothers, fathers’ mental health affects bonding, where paternal anxiety and parenting stress is related to bonding (Bieleninik et al., 2021). As mental health and parental cognitions affect bonding, parents within a couple might differ in their bonding to their infant, not least of their individual differences in mental health and cognitions.

### The present study

Summarized, bonding can be affected by repetitive negative thinking during pregnancy (Müller et al., 2013; Schmidt et al., 2017) in mothers. We do not know if this holds true for fathers too. We do not know if parents’ own cognitions and mental health can explain differences in bonding within couples, even though they are bonding to the same infant. Further, parental perception of their own infant’s temperament can be affected by difficulties in the parent themselves or the parent-infant relationship (MacKenzie and McDonough, 2009; Davies et al., 2021). Thus, a closer look at the effect of bonding and parents’ cognitions is warranted.

The sample was a resourceful one with high socioeconomic status and low levels of depressive symptoms. We asked; do repetitive negative thinking, attentional bias, and implicit attitudes during pregnancy predict bonding after birth? Do mothers and fathers differ in this regard? If so, would cognitions and depressive symptoms in one parent cause differences in bonding within a couple? Are parents’ perception of their infant’s temperament affected by bonding, depressive symptoms, cognitions, or the infants’ actual regularity?

Specifically, we hypothesized that a) higher levels of repetitive negative thinking, bias towards sad infant faces, and negative implicit associations to infants would lead to lower levels of bonding. We expected that b) parity would have a positive effect on bonding for mothers but not for fathers, and that c) education would not be a significant predictor for either, especially because of the low variance in the sample. Further, we expected that d) disparity in depressive symptoms or cognitions within a couple would predict disparity in bonding to their infant. Regarding the parental perception of infant temperament, we hypothesized that e) higher levels of bonding would lead to perceiving the infant as less difficult, while f) depressive symptoms and negative cognitions would have the opposite effect.

## Materials and methods

The present study was part of the Northern Babies Longitudinal Study (NorBaby; Høifødt et al., 2017), taking place in Northern Norway. Participants in this study were followed throughout the pregnancy until the infant was about 7 months old (last assessment was sent 6.5 months postnatally). There were six assessments, three during pregnancy (T1-T3) and three after birth (T4-T6). The present study applied data from T1, T4 and T5. T1 was completed between week 13–39 of gestation (*mean* 23.0, *median* 23, *SD* 3.62). The wide range is due to late recruitment of some participants. When they were recruited late, we prioritized the T1 assessment as this contained all demographic information and skipped T2 (and T3) when there was not enough time. Standard routine was to answer T1 at recruitment, T2 between week 24–30 of gestation, and T3 after week 31 of gestation, preferably with at least 4 weeks between assessments. T4 was sent to participants at week 6 after birth and completed between week 6–15 after birth (*mean* 8.17, *median* 7.71, *SD* = 1.96), and T5 was sent at week 16 after birth and completed between week 16–39 (4–9 months) after birth (*mean week* 21.25, *median week* 20.43, *SD* 3.49).

### Power calculations

Sample size was *a priori* estimated for the NorBaby study, see Høifødt et al. (2017) for details. Given feasibility and available resources, we aimed to recruit at least 200 families. We did not perform an *a priori* power calculation for the specifics of this analysis.

### Participants

350 participants were recruited to the NorBaby-study, 220 pregnant women and 130 partners (one female). The sample was a resourceful one, where the majority had higher education, good incomes, and experienced social support from family and friends (see Table 1 for details).

**Table 1:** Descriptives for T4 and T5 participants.

Variable	*T4*		*T5*	
		*N*		*N*
Men / Women	89/185	274	72/172	244
Age	*M* = 31.95 (*SD* = 4.9, range 20–49)	274	*M* = 31.91 (*SD* = 4.8, range 20–49)	244
Primiparous	52.2%	274	52.0%	244
Higher education	87.2%	274	86.9%	243
High income	71.4%	273	69.2%	242
Family support	91.6%	274	91.4%	244
Friend support	90.5%	274	90.2%	244

Higher education = university or college degree, bachelor level or higher. High income = average (750,000 NOK - 1,000,000 NOK) or high (> 1,000,000 NOK) per household. Family/Friend support = yes or no to the questions “Do you have enough family/friends to support you?”

### Procedure and measures

Participants were recruited through midwife-services both in the commune and at the University Hospital. All pregnant women and expecting partners living in the commune of Tromso who spoke Norwegian were eligible to participate. All who volunteered were included. Midwives asked the expecting mothers if they were interested in knowing more about the study and they were given a pamphlet to fill out if they were. Pamphlets were collected by the research team, and interested women were phoned to give more information about the study and invite them to participate. If they agreed to participate, they were invited to the first assessment either at the university or somewhere more convenient to them. Recruitment took place from September 2015 to October 2017.

The first assessment was completed in person with a member of the research group present. After that T2-T4 were sent to participants *via* e-mail. T5 and T6 was also sent by e-mail, but they also met in person as these assessments included filming and neuropsychological testing (not relevant for the present article). Measures relevant for the present article will be presented below.

#### Prenatal assessments (T1)

##### Demographics

Participants answered demographic questions at T1, including if they already had children and their level of education. Education was measured ordinally, from low (did not finish high school) to very high (more than 5 years at university/college). Previous depressive episodes were also reported, however this variable was not included in present analyses (please see Bohne et al., 2022 for analyses including history of depression).

##### The perseverative thinking questionnaire

To measure repetitive negative thinking we administered the Perseverative Thinking Questionnaire (PTQ; Ehring et al., 2011). This is a transdiagnostic questionnaire that measures if thoughts are repetitive, intrusive, and difficult to disengage from, if they are perceived unproductive and capture mental capacity. The questionnaire consists of 15 items that are statements about one’s thoughts, e.g., “The same thoughts keep going through my mind again and again.” Answer options range from 0 (never) to 4 (almost always), giving a total range of 0–60 where higher scores indicate higher degrees of repetitive negative thinking. Internal consistency was excellent both in the original version (α = 0.95) and in the present sample (MacDonald’s ω = 0.948).

##### Emotional dot-probe task

This is a modified version of the dot-probe task (MacLeod et al., 1986). We applied it to measure attentional bias to infant faces. The task presents participants with images of sad, happy, and neutral infant faces one at a time, on either the right or left side of the screen. Images are followed by an x on either the same or the opposite side of the screen. Participants have then to press a key (either “E” for left side or “I” for right side) to indicate where the x appeared. Response time is recorded. If participants respond faster on congruent trials (x appears on the same side of the screen as the stimulus image) than on incongruent trials (x appears on the opposite side), then the stimulus image caught their attention and they disengaged more slowly. Reaction times are calculated for each emotion (happy, sad, neutral infants), and compared. The task was completed at T1. Images were taken from the Tromso infant face database (Maack et al., 2017). Previous research demonstrated that a depressed group of expecting mothers differed from a non-depressed group of expecting mothers mainly on bias to sad faces (Bohne et al., 2021), and therefore we included only bias to sad faces in our analyses.

##### Single category implicit association test

The Single Category Implicit Association Test (SC-IAT; Karpinski and Steinman, 2006) is a modified version of the Implicit Association Test (Greenwald et al., 1998), here applied as a measure of implicit associations to neutral infant faces (image stimuli). The faces are taken from the Tromso infant face database (Maack et al., 2017). In the test, participants were asked to categorize words or infant faces as either positive or negative. This was done by sorting them either to the left or the right side of the screen using the “E” and “I” key. Stimuli (words or infant faces) were presented in random order on the screen, one at a time. There were two conditions, one where infants were sorted to the same side as positive words, and one where they were sorted with the negative words. Order of conditions were randomized. Response time was measured, and the difference between conditions is seen as a measure of implicit associations. There is a positive association towards infants when the response time is shorter for sorting the infant faces to the same side as the positive words. There is a negative association towards infants when the response time is shorter for sorting the infant faces to the same side as the negative words. The task was completed at T1.

##### Edinburgh postnatal depression scale

The scale was developed to screen for depressive symptoms in the perinatal period (Cox et al., 1987). It consists of 10 items assessing common depressive symptoms, e.g., “In the past 7 days, I have been so unhappy that I have had difficulty sleeping.” Each item is scored from 0–3 points and total scores range from 0–30 points. According to Norwegian validation and prevalence studies, a cutoff score ≥ 10 indicates possible depression and provides high sensitivity (Eberhard-Gran et al., 2001; Glavin et al., 2009). Even though it was originally developed to measure postnatal depression, it is widely used throughout the perinatal period, and validated for prenatal use as well (Field, 2017; Lydsdottir et al., 2019). Originally, the internal consistency of the scale was good (α = 0.87) and this was also the case in the present sample (MacDonald’s ω = 0.802).

#### Postnatal assessments (T4 and T5)

##### Edinburgh postnatal depression scale

As described above. Internal consistency at T4 was acceptable (MacDonald’s ω = 0.790).

##### Diurnal clock

To measure infant regulatory problems, we extracted data from diurnal clocks filled out by the parents at T4. The Diurnal Clock is a diary where participants registered their infants’ daily rhythm and behavioral state for two days and two nights. Diurnal clocks were coded according to criteria presented in Table 2. As many infants struggle with their night sleep at this age, a regulatory problem was not considered present unless they had a score of three or more.

**Table 2:** Criteria for coding of the Diurnal Clocks.

	Definition of when a regulatory problem is present
Total sleep	< 12 h during the 24 h
Night sleep	< 5 h continuous sleep between 23–06. Awakenings of 15 min or less is not counted since discontinued sleep for feeding during the night is considered normal.
Difficult to soothe	≥ 2 h of continuous fuzziness and/or crying
Excessive crying	≥ 3 h of crying during the 24 h

##### Maternal/paternal postnatal attachment scale

The scale is a measure of parental bonding (Condon and Corkindale, 1998; Condon et al., 2008). It consists of 19 items concerning parents’ thoughts and feelings towards their infant. Items are rated either on 2-, 3-, 4-or 5-point scales, and all items are therefore recoded to scores from 1 (poor bonding) to 5 (strong bonding) to ensure equal weighting of the items. This gives a total range from 19–95. Originally, internal consistency of both the MPAS and the PPAS was good, with Cronbach’s α varying from 0.78–0.81 depending on infant age (Condon and Corkindale, 1998; Condon et al., 2008). In the present sample, MacDonald’s ω_MPAS_ = 0.854, ω_PPAS_ = 0.842 indicating good consistency. The scale was answered at both T4 and T5, of interest here is the T4 score.

##### Parenting stress index – Child domain

The parenting stress index (PSI; Abidin, 1983) measures the level of stress parents experience in their parental role. The index has two subdomains, the parent domain (PD) and the child domain (CD). While the PD measures the stress experienced related to being a parent, the CD measures the stress related to child behavior. The present study included the CD only. A higher score on the child domain reflects more negative perceptions of their child’s characteristics and behavior, meaning the infant is perceived as having a more difficult temperament. Although not designed as a measure of temperament, the PSI-CD measures characteristics typically described as infant temperament (adaptability, mood, demandingness, distractability/hyperactivity, acceptability and reinforces parent; Olafsen et al., 2018). The PSI-CD consists of 47 items scored on a Likert scale from 1–5, giving a total range of 47–235. While other questionnaires designed to specifically measure infant temperament have struggled with poor or questionable internal consistency (Olafsen et al., 2018; Landsem et al., 2020), the PSI-CD in the present sample had excellent internal consistency (MacDonald’s ω = 0.91), as it also did originally (Cronbach’s α = 0.90 Abidin, 1995). The scale was completed at T5.

### Primary data analyses

Analyses were planned and pre-registered on the Open Science Framework before cleaning and analyses of the data begun (https://osf.io/dw3zs). In accordance with the plan, we conducted regressions to investigate parental cognitions during pregnancy as predictors of bonding after birth (MPAS/PPAS). We investigated bonding along with prenatal cognitions as predictors of the child domain of the parenting stress index (PSI-CD) at T5, while controlling for depressive symptoms and infant regulatory problems. The measure of bonding (MPAS and PPAS) is not the same questionnaire for mothers and fathers, although comparable. We ran analyses separately for mothers and fathers. Finally, we conducted a regression model to examine predictors of discrepancy in bonding within couples. Analyses were performed in Jasp (JASP, 2020) and R (R Core Team, 2020).

## Results

### Sample

There was some attrition during the study. At T4, 43 participants had resigned, while 33 participants missed this assessment. At T5, another 19 had resigned and 44 participants missed the assessment. This left us with 274 participants at T4 and 244 at T5. Comparing the missing group at T4 with the participating group revealed that fathers were more likely than mothers to drop out of the study (*χ^2^*(349) = 9.659, *p* = 0.002, Cramer’s *V* = 0.166). The missing group had 52% fathers, while the participating group had 32%. The missing group at T4 was also less educated than the participating group (*t*(346) = 5.200, *p* < 0.001, Cohen’s *d* = 0.681). The groups did not differ on the level of income (*t*(345) = 1.820, *p* = 0.070, *d* = 0.238).

Mothers and fathers differed significantly on several measures (see Table 3).

**Table 3:** Difference between mothers and fathers.

Measures	Mothers Mean (SD)	*N*	Fathers Mean (SD)	*N*	*t*	*p*	*d*
*T1 measures*							
Repetitive negative thinking	17.246 (10.280)	179	12.791 (9.624)	84	3.450	**< 0.001**	0.447
Attentional bias	−7.249 (15.645)	171	−9.301 (15.490)	84	0.991	0.323	0.132
Implicit associations	0.066 (0.295)	171	0.032 (0.248)	84	0.965	0.336	0.125
*T4 measures*							
Bonding	4.323 (0.419)	180	4.025 (0.378)	88	5.839	**< 0.001**	0.746
Prenatal depressive symptoms	4.475 (3.597)	185	2.534 (2.840)	89	3.042	**0.003**	0.392
*T5 measures*							
Infant temperament	1.831 (0.359)	169	1.919 (0.293)	71	1.816	0.071	0.257

T-tests on T1 measures is run with participants from T4. Repetitive negative thinking = PTQ at T1, Implicit associations as measured by the Single Category Implicit Association Task, Attentional bias = bias to sad infant faces as measured by Emotional Dot-Probe Task, Prenatal depressive symptoms = EPDS at T1, Bonding = MPAS or PPAS at T4, Infant temperament = PSI-CD at T5. Bold values are significant at 0.05 level.

### Predictors of bonding

A hierarchical regression model predicting parent-infant bonding (as measured by MPAS and PPAS at T4) was applied (Table 4). The demographic variables parity and education were entered in step 1. Cognitive variables and prenatal depressive symptoms were entered in step 2.

**Table 4:** Results of hierarchical regression predicting bonding in mothers and fathers.

	Mothers				Fathers			
Predictor variables	B	*β (95% CI)*	*t*	*p*	B	*β (95% CI)*	*t*	*p*
**Model 1**								
Parity	0.186	0.222 (0.068, 0.376)	2.842	**0.005**	−0.140	−0.181 (−0.399, 0.036)	−1.659	0.101
Education	−0.052	−0.086 (−0.240, 0.068)	−1.100	0.273	−0.139	−0.278 (−0.494, −0.060)	−2.539	**0.013**
**Model 2**								
Parity	0.139	0.166 (0.017, 0.314)	2.195	**0.030**	−0.162	−0.210 (−0.412, −0.008)	−2.067	**0.042**
Education	−0.056	−0.093 (−0.245, 0.060)	−1.207	0.229	−0.145	−0.289 (−0.494, −0.084)	−2.818	**0.006**
Repetitive negative thinking	−0.010	−0.244 (−0.406, −0.072)	−2.808	**0.006**	−0.010	−0.249 (−0.514, −0.001)	−1.846	0.069
Implicit associations	0.206	0.146 (−0.001, 0.293)	1.960	0.052	−0.274	−0.173 (−0.378, 0.033)	−1.671	0.099
Attentional bias	0.000	0.006 (−0.144, 0.144)	0.078	0.938	−0.001	−0.044 (−0.245, 0.163)	−0.424	0.673
Prenatal depressive symptoms	−0.017	−0.139 (−0.320, 0.042)	−1.507	0.134	−0.024	−0.182 (−0.451, 0.090)	−1.341	0.184

B = Unstandardized beta, β = Standardized beta. Repetitive negative thinking = PTQ at T1, Implicit associations as measured by the Single Category Implicit Association Task, Attentional bias = bias to sad infant faces as measured by Emotional Dot-Probe Task, Prenatal depressive symptoms = EPDS at T1. Model 1 (mothers): R = 0.226, R^2^ = 0.051, Adjusted R^2^ = 0.039, Standard error = 0.412. Model 2 (mothers): R = 0.440, R^2^ = 0.194, Adjusted R^2^ = 0.162, Standard error = 0.385. Model 1 (fathers): R = 0.317, R^2^ = 0.100, Adjusted R^2^ = 0.077, Standard error = 0.371. Model 2 (fathers): R = 0.525, R^2^ = 0.276, Adjusted R^2^ = 0.215, Standard error = 0.342. Bold values are significant at 0.05 level.

For mothers, model 1 was significant, explaining 3.9% of the variance (*F*(2, 159) = 4.268, *p* = 0.016). As predicted in hypothesis b) parity came out as a significant predictor, where having children from before was positively associated with bonding. Model 2 explained 16.2% of the variance (*F*(6,155) = 6.203, *p* < 0.001), and in addition to parity, repetitive negative thinking was a significant predictor. Implicit associations came close to statistical significance (*p* = 0.052) and had an effect size like that for parity (ß = 0.146 vs. ß = 0.166, see Table 4). Attentional bias was not a significant predictor. Hypothesis a) was partly supported in mothers.

**Figure 1:**
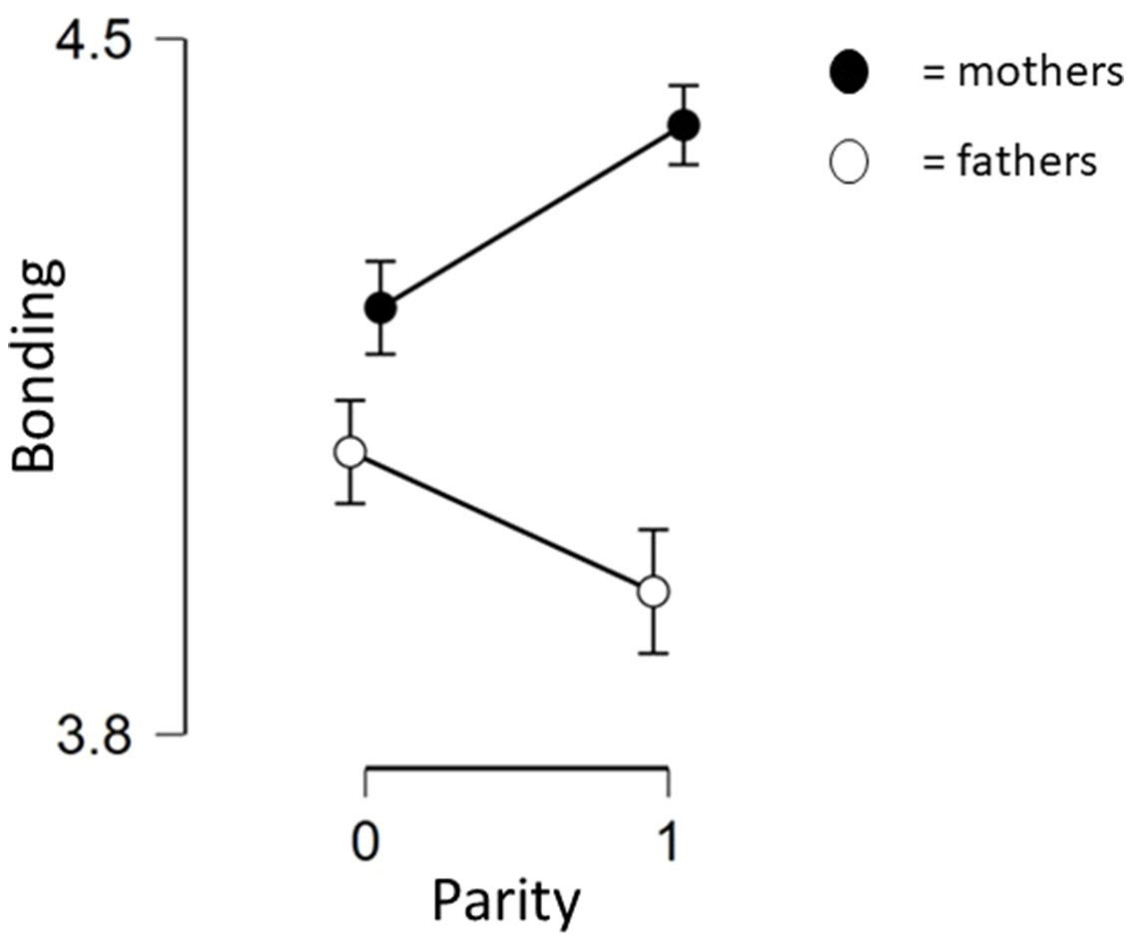
Different effect of parity on bonding for mothers and fathers. Bonding as measured by Maternal/Paternal Postnatal Attachment Scale, mean scores. Parity is either yes or no.

For fathers, model 1 explained 7.7% of the variance (*F*(2, 76) = 4.239, *p* = 0.018) and education was significantly and negatively predicting bonding. Less educated fathers had higher levels of father-infant bonding, refuting hypothesis c). Model 2 explained 21.5% of the variance (*F*(6, 72) = 4.569, *p* < 0.001) and both education and parity were significant. In line with hypothesis b) the effect of parity was opposite from mothers, as it was negatively associated with bonding for fathers (see Figure 1). Repetitive negative thinking was not significant for fathers (*p* = 0.069), although there was a trend in the same direction as for mothers, and the effect size (ß = −0.249) was similar to that for parity and education (Table 4).

#### Difference in bonding within couples

**Figure 2:**
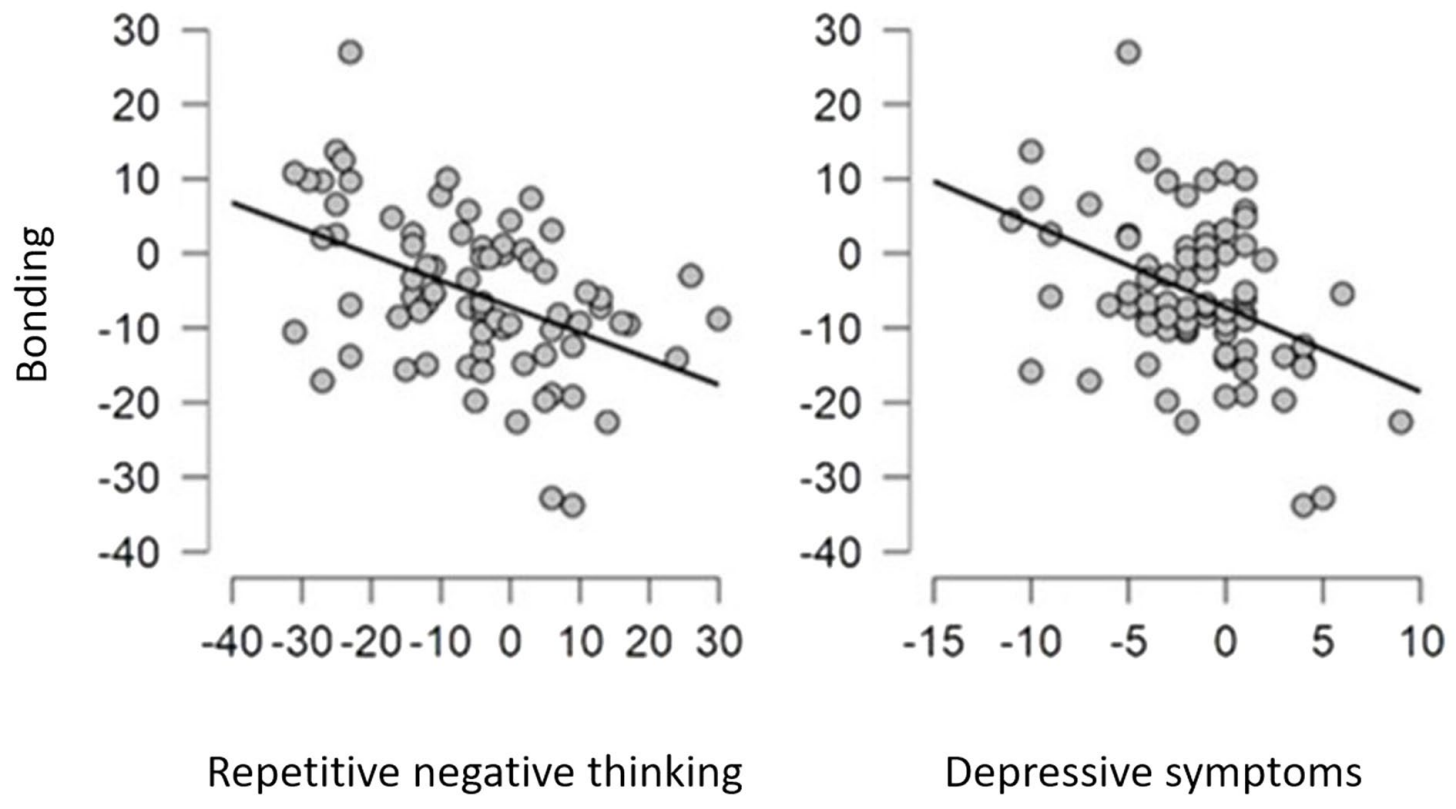
Plots of associations between difference in bonding within couples and the difference in repetitive negative thinking and depressive symptoms.

Parents can differ in their amount of bonding to their infant, so we looked at whether this difference was due to differences in their cognitions and depressive symptoms. To investigate what predicted higher discrepancy in bonding within couples, we extracted participants where both mother and father had answered both T1 and T4. This left us with 79 couples. We calculated the difference in scores within each couple, subtracting mothers’ scores from fathers’ scores. The variables included as predictors were education, prenatal assessed implicit associations and repetitive negative thinking, and postnatal depressive symptoms. Due to power, we excluded attentional bias as this was not significant for bonding in either mothers or fathers, and parity as only 12 couples differed within themselves on number of children. We ran a regression analysis with the difference in bonding as outcome (see SOM Supplementary Table 6). The model was significant (*F*(4,74) = 8.062, *p* < 0.001) and explained 26.6% of the variance. Within couples, difference in repetitive negative thinking and postnatal depressive symptoms were significant predictors for the difference in bonding, supporting hypothesis d). Higher discrepancy in bonding within a couple is in part explained by higher levels of prenatal repetitive negative thinking or postnatal depressive symptoms in one of the parents, as can be seen in Figure 2. Differences in education or implicit associations within a couple did not explain differences in bonding.

### Predictors of parents’ perception of infant temperament

A hierarchical regression model predicting parents’ perception and experience of their infant temperament (measured by the child domain of the parenting stress index (PSI-CD) at T5) was applied for mothers and fathers separately (see Table 5). Due to power and the results for bonding, attentional bias and implicit associations were excluded from this analysis. The only prenatal cognitive factor repetitive negative thinking was entered in step 1 (see SOM for a model including the implicit association, and a model including education and parity, only for mothers). In step 2, variables from T4 were entered: bonding, postnatal depressive symptoms, and infant regulatory problems.

**Table 5:** Results of hierarchical regression predicting parents’ perception of infant temperament (PSI-CD T5.)

Predictor variables	Mothers				Fathers			
	*B*	*β (95% CI)*	*t*	*p*	*B*	*β (95% CI)*	*t*	*p*
**Model 1**								
Repetitive negative thinking	0.006	0.149 (−0.027, 0.320)	1.674	0.097	0.006	0.248 (−0.002, 0.502)	1.826	0.074
**Model 2**								
Repetitive negative thinking	−0.001	−0.039 (−0.213, 0.133)	−0.448	0.655	0.000	0.007 (−0.270, 0.309)	0.046	0.963
Bonding	−0.301	−0.384 (−0.568, −0.199)	−4.118	**< 0.001**	−0.163	−0.230 (−0.509, 0.048)	−1.662	0.103
Infant regularity	0.078	0.089 (−0.072, 0.249)	1.095	0.276	−0.030	−0.042 (−0.306, 0.222)	−0.320	0.751
Postnatal depressive symptoms	0.017	0.167 (−0.029, 0.358)	1.723	0.087	0.040	0.387 (0.107, 0.663)	2.821	**0.007**

B = Unstandardized beta, β = Standardized beta. Repetitive negative thinking = PTQ at T1, Bonding = MPAS/PPAS at T4, Infant regularity as measured with diurnal clocks at T4, Postnatal depressive symptoms = EPDS at T4. Model 1 (mothers): R = 0.149, R^2^ = 0.022, Adjusted R^2^ = 0.014, Standard error = 0.319. Model 2 (mothers): R = 0.494, R^2^ = 0.244, Adjusted R^2^ = 0.218, Standard error = 0.284. Model 1 (fathers): R = 0.248, R^2^ = 0.061, Adjusted R^2^ = 0.043, Standard error = 0.279. Model 2 (fathers): R = 0.511, R^2^ = 0.261, Adjusted R^2^ = 0.200, Standard error = 0.255. Bold values are significant at 0.05 level.

For mothers, model 1 was not significant (*F*(1, 123) = 2.802, *p* = 0.097), but model 2 was (*F*(4, 120) = 9.663, *p* < 0.001). Model 2 explained 21.8% of the variance, and bonding was the only significant predictor. The more bonding, the less stressful the infant was experienced. This supported hypothesis e), however as depressive symptoms and negative thinking were not significant, hypothesis f) was not supported for mothers.

For fathers, hypothesis f) was confirmed, with postnatal depressive symptoms being the only significant predictor in model 2 (*F*(4,48) = 4.244, *p* = 0.005), explaining 20% of the variance. Higher levels of depressive symptoms predicted more stressful experience of the infant. Bonding was not significant for fathers, not supporting hypothesis e) for fathers.

#### Indirect effect of repetitive negative thinking

**Figure 3:**
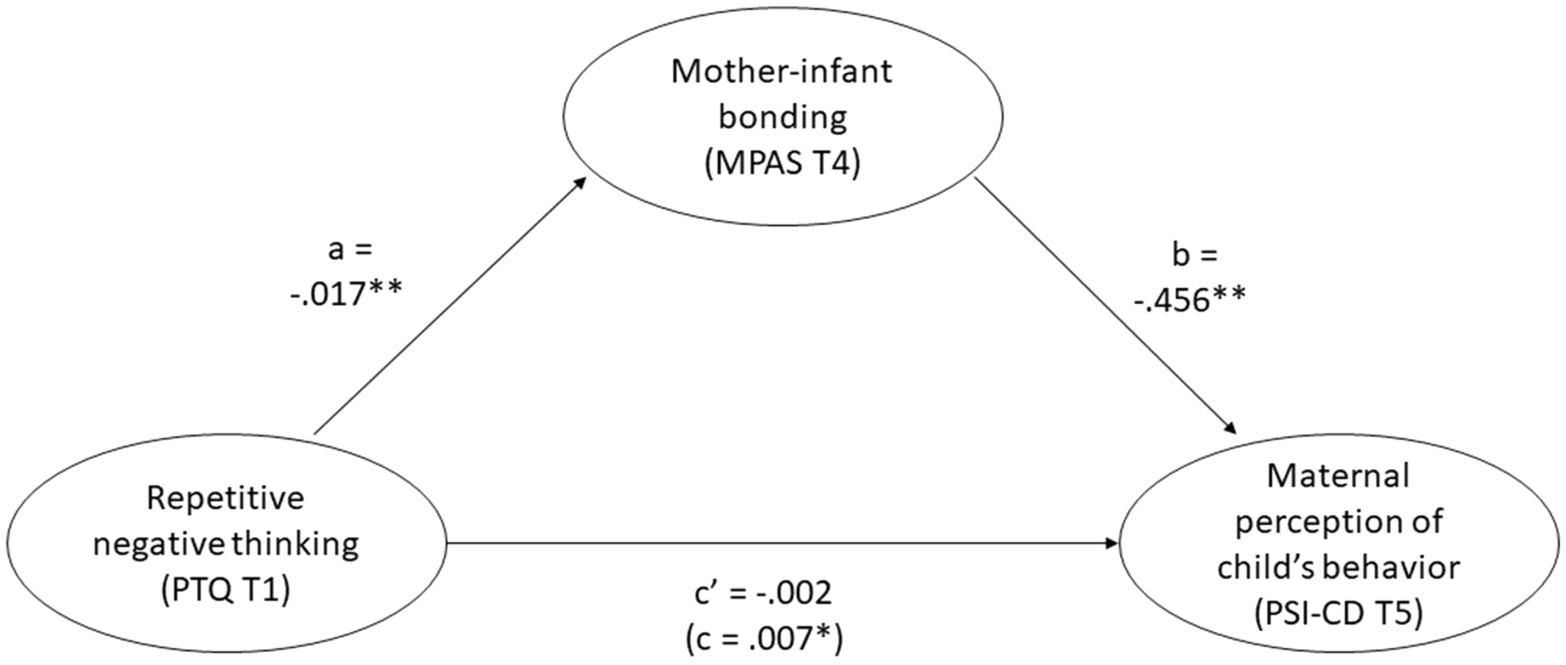
Indirect effect of repetitive negative thinking. Coefficients are unstandardized. a and b are the indirect path, c’ is the direct path. Total effect (c) in brackets. * = *p* < 0.05, ** = *p* < 0.001.

As repetitive negative thinking during pregnancy was a strong predictor of bonding after birth for mothers, but not for the mothers’ perception and experience of infant temperament, we decided to examine if repetitive negative thinking had an indirect effect on the perception of the infant through bonding. The mediation analysis confirmed our assumption: the effect of repetitive negative thinking on mothers’ perception of their infant’s temperament, was fully mediated by mother-infant bonding (see Figure 3). Repetitive negative thinking affects bonding, and then bonding affects the perception of the infant’s temperament.

## Discussion

The present study investigated if parental cognitions during pregnancy predicted bonding after birth in a resourceful and low depression sample. The findings partly support the role of cognitions on bonding. Repetitive negative thinking during pregnancy was a significant predictor of bonding after birth in mothers, and there was a similar trend for fathers. However, attentional bias and implicit associations to infant faces were not related to bonding, thereby only partly confirming hypothesis a). Notably, there were differences between mothers and fathers in the predictors of bonding. As hypothesized, b) parity was a significant predictor for both mothers and fathers, and in opposite directions. For mothers, parity was positively related to bonding, suggesting that experience with mothering had a positive effect. For fathers, parity was negatively related to bonding, which was in line with previous findings where fathers bond more strongly to their firstborn (de Cock et al., 2016). Further, hypothesis c) was partly confirmed, education had no significant effect on mothers’ bonding, whereas fathers’ education level was negatively related to bonding.

Regarding parents’ perception of their infant’s temperament (PSI-CD at T5), we found partly support for our hypotheses. After controlling for infant regulatory problems, for mothers, bonding (T4) was the only significant predictor, and for fathers, depressive symptoms (T4) were the only significant predictor. We found that there was an effect of repetitive negative thinking on mothers’ perception of their infant’s temperament that was fully mediated by bonding, meaning that repetitive negative thinking affects bonding, which further affects mothers’ perception of infant temperament.

### Parental cognitions

#### Repetitive negative thinking

In line with the model of DeJong et al. (2016), the present study illustrated that repetitive negative thinking in mothers can have a negative effect on maternal bonding. One can easily imagine how the pregnancy itself can fuel repetitive negative thoughts, worrying about both the infant and how the parental role and the new life will be. Having a baby can be overwhelming for anyone, and if you are prone to a repetitive negative thinking style, this may increase the burden. As the baby can be the source of many stressful thoughts, this may challenge the bonding process. As Stein et al. (2009, 2014) stated, being preoccupied with negative thoughts challenges the parents’ ability to be present, sensitive, responsive, and focused on the infant’s cues, and may thereby also preclude the bonding process. Although not statistically significant, there was a trend in the same direction for fathers and changing this thinking style is likely to be beneficial for new fathers too.

#### Implicit associations and attentional bias to infant faces

Although the implicit association score was not a statistically significant predictor for bonding in mothers, its effect size was comparable to that for parity and thus implicit associations might play a role. Further research is needed to understand what leads to negative associations to infants, and if this can affect parenting. The attentional bias to sad faces was not significant and had negligible effect sizes in the model for mothers and the model for fathers. Behavioral tasks may not be suited to predict individual differences (Dang et al., 2020). In addition, both the SC-IAT and the emotional dot probe task have been criticized for low reliability (Staugaard, 2009; Chevance et al., 2017), and may be better suited for comparing groups (Staugaard, 2009; Bohne et al., 2021). Further, behavioral tasks and self-report measures are often only weakly correlated (Dang et al., 2020). Thus, questionnaires that measure cognitive styles might be more potent than experimental tests when looking into the effect of parental cognitions on their parenting experiences.

### Differences between mothers and fathers

The present study revealed some interesting differences between mothers’ and fathers’ cognitions in the perinatal period. While mothers had more repetitive negative thinking during pregnancy and more depressive symptoms after birth, they still experienced stronger bonding to their infant than fathers. This may of course be explained by biological factors, breast feeding, and the amount of time the mother spends with her newborn. Even so, this tells us that fathers might need to put a larger effort in the bonding process to reach the same level as the mothers. In Norway, fathers have a mandatory paternity leave (it was 10–15 weeks at the time of data collection). It would be interesting to measure bonding after this period to see if the difference would be equalized. Schaber et al. (2021) found an unstable effect of duration of paternity leave on bonding though, leaving the question open. The authors suggest other factors like partner satisfaction might be more important for paternal bonding, as it indicates the ability to form good relationships (Schaber et al., 2021).

In line with previous research (de Cock et al., 2016), mothers bonded more strongly when it was not their first child, while the reverse applied for fathers. Again, the time spent with the newborn may be of essence. When it is their first child, both parents can fully attend to the newborn, while when it is the second (or third, or fourth), other children present also demand attention. As the mother is the primary caregiver during the first months after birth, naturally the father attends more to the other children. One could imagine the father even bonding more strongly to the older siblings in this transition, while the mother cares for the newborn. We did not assess this but recommend future studies to investigate the dynamics of the entire family.

#### Education

Interestingly, education was a significant predictor of bonding in fathers, where lower educated fathers had stronger bonding to their infant. This was in line with previous studies on mothers’ bonding (Kinsey et al., 2014; Cuijlits et al., 2019). One explanation for this could be as Kinsey et al. (2014) suggested, that higher educated fathers are less biased by social desirability and therefore, more honest about their feelings towards the infant. However, we find it likely that higher educated fathers are more likely to have demanding jobs, and therefore might have less capacity for bonding. Just as repetitive negative thoughts can keep you occupied, worry, planning, or problems solving related to work can keep you occupied as well.

### Infant temperament

Bonding was predictive of mothers’ perceived infant temperament, which is in line with the cross-sectional findings of Davies et al. (2021). de Cock et al. (2016) also found that both mothers and fathers with low levels of bonding reported more difficult temperament in their infant at 6 months. In the present study, depressive symptoms, but not bonding, were predictive of perceived infant temperament for fathers. It seems their own well-being colors how they see their infant, while for mothers, the relationship to their infant is essential for how they experience their infant’s temperament. As there might be a discrepancy between actual infant behavior and perceived infant temperament (MacKenzie and McDonough, 2009), we encourage more research that measures both. In the present study, regulation problems in the infant were not significantly related to later perception of infant temperament.

### Discrepancy within couples

As mothers and fathers within a couple bond to the same infant, the difference in bonding between mother and father must be caused by something else than infant behavior. To our knowledge, our study is the first to investigate discrepancies in bonding levels within couples. Perhaps this is because previous findings indicate that couples mostly display comparable levels of bonding (de Cock et al., 2016). Our analysis suggests that when the difference in bonding within a couple was large, one of the parents experienced higher levels of either repetitive negative thinking or depressive symptoms than the other parent. However, the amount of time spent with the child and other probable factors were not measured, and so one must be careful to conclude. Even so, if one parent is struggling on a personal level with depressive symptoms or negative thoughts, and the other one is bonding strongly to their infant, the healthy parent will likely spend the most time with the infant. This could turn into a vicious cycle, where the struggling parent is prevented from building a stronger bond to the infant, thereby delaying both the recovery and the bonding.

### Implications for health care services

Bonding is predictive of child outcome (Alhusen et al., 2013; Le Bas et al., 2020), parenting stress (de Cock et al., 2017), and, as the present study has shown; parents’ perceived infant temperament. Therefore, bonding is an important aspect to be aware of when providing health care during the perinatal period. Already during pregnancy, cognitive thinking styles like repetitive negative thinking, shown here to have a negative effect on bonding, can be identified. This gives the opportunity to intervene before birth, and thereby enhance bonding and a good start for the new family. There is a range of therapeutic interventions that target such thinking styles (see Monteregge et al., 2020 for a meta-analysis), and also interventions more specific for this group, e.g., mindful parenting interventions (Potharst et al., 2017).

Health professionals should strive to screen parents’ thoughts and emotions during pregnancy and offer intervention when applicable. When parents seek help because of regulatory problems in the infant, health professionals must keep in mind that the parents’ mental health is equally important to screen as is the infant’s behavior.

Families expecting their second or third child might benefit from information that fathers bond more easily to their first child, so that families can be aware and facilitate paternal bonding to a higher degree.

### Limitations

There was some attrition between assessments. More fathers and less educated participants dropped out. This might have affected the results. However, education still came out as a significant predictor of bonding in fathers even though a large majority had higher education. In retrospect, it would have been beneficial to have kept fathers from dropping out, and future studies should apply a different data collection strategy to mitigate this. For example, instead of having mothers and their infant visit the university during work hours for their T5 assessment, home visits after work could be made to ensure father participation as well. Still, it was a clear strength that we included fathers, which allowed us to identify differences between mothers and fathers.

The present sample was a resourceful one, where most parents were mentally healthy, highly educated, and had a good income. We therefore cannot generalize to more vulnerable groups. Future research should target groups with lower socioeconomic status and examine how worries about economy and employment might affect bonding as it would easily occupy mental capacity. Such worries are related to increased distress in other contexts (e.g., Mækelæ et al., 2021), and so it is probable that it would affect bonding as well.

The present study did not consider partner satisfaction or adult attachment style, which previous studies have suggested as an important predictor of bonding (Nordahl et al., 2020; Schaber et al., 2021). The ability to form close relationships may be an individual characteristic affecting both parent–child relations and romantic relations. Repetitive negative thinking or rumination is negatively affecting bonding (this study), and romantic relations (Jostmann et al., 2011; Elphinston et al., 2013). This indicates that a negative thinking style may be a mechanism involved in relationship difficulties in general.

Due to our sample size, especially fathers, we did not investigate possible interaction effects. It would be interesting to examine, e.g., if the effect of parity in fathers interact with the effect of education. Fathers with lower education might bond stronger to their first-born whereas fathers with higher education might experience a weaker effect of parity. Future research should address possible interaction effects.

## Conclusion

The present study investigated parental cognitions during pregnancy and their effect on bonding after birth. What affected bonding differed between mothers and fathers. In fathers, bonding was strongest if it was their first child and if they had lower education. In mothers, repetitive negative thinking during pregnancy negatively affected bonding whereas parity affected bonding positively. Further, maternal bonding affected how the mother perceived her infant’s temperament. Attentional bias and implicit attitudes did not affect bonding.

Bonding quality is related to child development outcome. Beneficial factors for bonding quality should be strengthened and detrimental factors should be debilitated when possible. Identifying repetitive negative thoughts during pregnancy and helping reduce these thoughts might therefore facilitate stronger bonding and a better start for the family. Health care services should strive to screen parents’ thoughts and feelings already during pregnancy and offer intervention where needed.

## Data availability statement

The datasets presented in this study can be found in online repositories. The names of the repository/repositories and accession number(s) can be found at: https://osf.io/dw3zs/?view_only=8a4961745d294904a29ce37e527a9932.

## Ethics statement

The study was approved by the Regional Committees for Medical and Health Research Ethics, ref. 2015/614. We confirm that the present study complies with the 1964 Declaration of Helsinki and later addenda. Written informed consent to participate in this study was provided by the participants. Participating parents gave consent on behalf of their infant.

## Author contributions

AB, RH, DN, and IL were involved in data acquisition. GP designed the cognitive task. GP and AB analyzed the data. AB drafted the work. All authors were involved in the conception and design, critically revised and approved for publishing.

## Conflict of interest

The authors declare that the research was conducted in the absence of any commercial or financial relationships that could be construed as a potential conflict of interest.

## Publisher’s note

All claims expressed in this article are solely those of the authors and do not necessarily represent those of their affiliated organizations, or those of the publisher, the editors and the reviewers. Any product that may be evaluated in this article, or claim that may be made by its manufacturer, is not guaranteed or endorsed by the publisher.

## Supplementary material

The Supplementary material for this article can be found online at: https://www.frontiersin.org/articles/10.3389/fpsyg.2022.986757/full#supplementary-material
