# Supplementary material for: Do parental cognitions during pregnancy predict bonding after birth in a low-risk sample?
Source: Front Psychol. 2022 Nov 14;13:986757. doi: 10.3389/fpsyg.2022.986757 (PMC9704052; doi:10.3389/fpsyg.2022.986757)
Supplement: Supplementary file 1 [file Data_Sheet_1.pdf]

Table 6

Results of regression predicting difference in bonding (MPAS/PPAS at T4) within couples

Predictor variables	B	*β (95% CI)*	*t*	*p*
Education (diff)	-1.734	-.146 (-.349, .057)	-1.431	.157
Repetitive negative thinking (diff)	-.272	-.358 (-.565, -.150)	-3.434	<.001
Implicit associations (diff)	-1.848	-.071 (-.273, .132)	-.694	.490
Postnatal depressive symptoms (diff)	-.846	-.305 (-.513, -.097)	-2.917	.005

Note. B = Unstandardized beta, β = Standardized beta. All differentials are calculated subtracting mothers' values from fathers' values. Education (diff) = difference in education within couples, Repetitive negative thinking (diff) = difference within couples on PTQ at T1, Implicit associations (diff) = difference within couples on the SC-IAT, Postnatal depressive symptoms (diff) = difference within couples on EPDS at T4. Model summary: R = .551, R^2 = .304, Adjusted R^2 = .266, Standard error = 8.933.

Table 7

Linear Regression_predicting mothers' report of infant temperament (PSI-CD; version incl sc-IAT)

Model Summary

Model	R	R²	Adjusted R²	RMSE	R² Change	F Change	df1	df2	p
0	0.154	0.024	0.007	0.324	0.024	1.439	2	118	0.241
1	0.524	0.275	0.243	0.283	0.251	13.270	3	115	< .001

Note. Null model includes PTQ_T1, T1_IAT_d

ANOVA

Model		Sum of Squares	df	Mean Square	F	p
0	Regression	0.303	2	0.151	1.439	0.241
	Residual	12.416	118	0.105		
	Total	12.719	120			
1	Regression	3.496	5	0.699	8.717	< .001
	Residual	9.223	115	0.080		
	Total	12.719	120			

Note. Null model includes PTQ_T1, T1_IAT_d

Coefficients

							95% CI	
Model		Unstandardized	Standard Error	Standardized	t	p	Lower	Upper
0	(Intercept)	1.728	0.064		27.023	< .001	1.602	1.855
	PTQ_T1	0.006	0.003	0.151	1.656	0.100	-0.001	0.012
	T1_IAT_d	0.048	0.100	0.043	0.477	0.634	-0.150	0.245
1	(Intercept)	3.194	0.348		9.185	< .001	2.505	3.883
	**T4_MPAS_mean**	-0.331	0.074	**-0.417**	-4.443	**< .001**	-0.478	-0.183
	T4_reg_prob_yes_no	0.089	0.074	0.100	1.206	0.230	-0.057	0.235
	EPDS_T4	0.018	0.010	0.173	1.769	0.079	-0.002	0.038
	PTQ_T1	-0.002	0.003	-0.052	-0.598	0.551	-0.008	0.005
	T1_IAT_d	0.096	0.089	0.088	1.084	0.281	-0.079	0.271

Descriptives

Coefficients

Model	Unstandardized	Standard Error	Standardized	t	p	95% CI Lower	95% CI Upper

	N	Mean	SD	SE
PSI_CD_T5_mean	121	1.824	0.326	0.030
T4_MPAS_mean	121	4.299	0.410	0.037
T4_reg_prob_yes_no	121	0.157	0.365	0.033
EPDS_T4	121	3.562	3.141	0.286
PTQ_T1	121	16.364	8.674	0.789
T1_IAT_d	121	0.063	0.298	0.027

Table 8

Linear Regression_ predicting mothers' report of infant temperament (PSI-CD; incl IAT, education and parity)

Model Summary

Model	R	R²	Adjusted R²	RMSE	R² Change	F Change	df1	df2	p
0	0.154	0.024	0.007	0.324	0.024	1.439	2	118	0.241
1	0.524	0.275	0.230	0.286	0.251	7.827	5	113	< .001

Note. Null model includes PTQ_T1, T1_IAT_d

ANOVA

Model		Sum of Squares	df	Mean Square	F	p
0	Regression	0.303	2	0.151	1.439	0.241
	Residual	12.416	118	0.105		
	Total	12.719	120			
1	Regression	3.497	7	0.500	6.121	< .001
	Residual	9.222	113	0.082		
	Total	12.719	120			

Note. Null model includes PTQ_T1, T1_IAT_d

Coefficients

Model		Unstandardized	Standard Error	Standardized	t	p	95% CI	
							Lower	Upper
0	(Intercept)	1.728	0.064		27.023	< .001	1.602	1.855
	PTQ_T1	0.006	0.003	0.151	1.656	0.100	-0.001	0.012
	T1_IAT_d	0.048	0.100	0.043	0.477	0.634	-0.150	0.245
1	(Intercept)	3.217	0.419		7.668	< .001	2.386	4.048
	**T4_MPAS_mean**	-0.332	0.077	**-0.418**	-4.332	**< .001**	-0.484	-0.180
	T4_reg_prob_yes_no	0.089	0.076	0.100	1.170	0.244	-0.062	0.240
	EPDS_T4	0.017	0.011	0.168	1.518	0.132	-0.005	0.040
	PTQ_T1	-0.002	0.003	-0.052	-0.583	0.561	-0.009	0.005
	T1_IAT_d	0.094	0.091	0.086	1.037	0.302	-0.086	0.275
	Education	-0.004	0.044	-0.008	-0.091	0.928	-0.091	0.083
	NotFirstChild	-0.004	0.055	-0.006	-0.071	0.943	-0.114	0.106

Descriptives

	N	Mean	SD	SE
PSI_CD_T5_mean	121	1.824	0.326	0.030
T4_MPAS_mean	121	4.299	0.410	0.037
T4_reg_prob_yes_no	121	0.157	0.365	0.033
EPDS_T4	121	3.562	3.141	0.286
PTQ_T1	121	16.364	8.674	0.789
T1_IAT_d	121	0.063	0.298	0.027
Education	121	3.545	0.671	0.061
NotFirstChild	121	0.504	0.502	0.046
